# Is Frailty Index a better predictor than pre-stroke modified Rankin Scale for neurocognitive outcomes 3-months post-stroke?

**DOI:** 10.1186/s12877-022-02840-y

**Published:** 2022-02-19

**Authors:** Ragnhild Munthe-Kaas, Stina Aam, Ingvild Saltvedt, Torgeir Bruun Wyller, Sarah T. Pendlebury, Stian Lydersen, Guri Hagberg, Till Schellhorn, Siri Rostoft, Hege Ihle-Hansen

**Affiliations:** 1 Department of Medicine, Bærum Hospital, Vestre Viken Hospital Trust, Drammen, Norway; 2 Institute of Clinical Medicine, University of Oslo, Oslo, Norway; 3 Department of Neuromedicine and Movement Science, Faculty of Medicine and Health Science, NTNU-Norwegian University of Science and Technology, Trondheim, Norway; 4 Department of Geriatric Medicine, Clinic of Medicine, St. Olavs Hospital, Trondheim University Hospital, Trondheim, Norway; 5 Department of Geriatric Medicine, Oslo University Hospital, Oslo, Norway; 6 Wolfson Centre for Prevention of Stroke and Dementia, Nuffield Department of Clinical Neurosciences, University Of Oxford, and the NIHR Biomedical Research Centre Oxford University Hospitals NHS Foundation Trust, Oxford, UK; 7 Departments of Acute Internal Medicine and Geratology, Oxford University Hospitals NHS Foundation Trust, Oxford, UK; 8 Department of Mental Health, Faculty of Medicine and Health Science, Regional Centre for Child and Youth Mental Health and Child Welfare, NTNU-Norwegian University of Science and Technology, Trondheim, Norway; 9 Department of Neurology, Oslo University Hospital, Oslo, Norway; 10 Division of Radiology and Nuclear Medicine, Oslo University Hospital, Oslo, Norway

**Keywords:** Stroke, Cognition, Cognitive Impairment, Frailty, Prediction

## Abstract

**Background:**

The prognostic value of frailty measures for post-stroke neurocognitive disorder (NCD) remains to be evaluated.

**Aims:**

The aim of this study was to compare the predictive value of pre-stroke FI with pre-stroke modified Rankin Scale (mRS) for post-stroke cognitive impairment. Further, we explored the added value of including FI in prediction models for cognitive prognosis post-stroke.

**Methods:**

We generated a 36-item Frailty Index (FI), based on the Rockwood FI, to measure frailty based on pre-stroke medical conditions recorded in the Nor-COAST multicentre prospective study baseline assessments. Consecutive participants with a FI score and completed cognitive test battery at three months were included. We generated Odds Ratio (OR) with NCD as the dependent variable. The predictors of primary interest were pre-stroke frailty and mRS. We also measured the predictive values of mRS and FI by the area (AUC) under the receiver operating characteristic curve.

**Results:**

598 participants (43.0% women, mean/SD age = 71.6/11.9, mean/SD education = 12.5/3.8, mean/SD pre-stroke mRS = 0.8/1.0, mean/SD GDS pre-stroke = 1.4/0.8, mean/SD NIHSS day 1 3/4), had a FI mean/SD score = 0.14/0.10. The logistic regression analyses showed that FI (OR 3.09), as well as the mRS (OR 2.21), were strong predictors of major NCD. When FI and mRS were entered as predictors simultaneously, the OR for mRS decreased relatively more than that for FI. AUC for NCD post-stroke was higher for FI than for mRS, both for major NCD (0.762 vs 0.677) and for any NCD (0.681 vs 0.638).

**Conclusions:**

FI is a stronger predictor of post-stroke NCD than pre-stroke mRS and could be a part of the prediction models for cognitive prognosis post-stroke.

**Trial Registration:**

ClinicalTrials.gov Identifier: NCT02650531.

**Supplementary Information:**

The online version contains supplementary material available at 10.1186/s12877-022-02840-y.

## Background

One out of four stroke survivors fulfills the criteria for major post-stroke neurocognitive disorder (NCD) after one year [1], and any post-stroke NCD is reported to be up to 53% in hospital-based studies[2].

Health status, disability and cognition pre-stroke are important prognostic factors in a stroke population. Predictors of different cognitive outcomes are needed to target interventions preventing cognitive decline, and to provide optimal care post-stroke. Vascular risk factors, stroke related factors, and frailty measures are addressed in existing prediction models of post-stroke NCD [3, 4].

People age biologically at different rates [5, 6]. Frailty is a condition of vulnerability associated with an increased risk of adverse health outcomes such as functional decline and mortality. A gradual decline in physiological reserves is expected with age, but in frailty, this decline is accelerated and occurs in various organ systems. The many scales for measuring frailty reflect the uncertainty regarding the best way to assess this syndrome [7]. There are, however, two main theories of the definition of frailty [5]. In the model by Fried and colleagues, the so-called physical frailty phenotype is described as a clinical syndrome [8]. Presence of three or more of the following symptoms: weight loss, weakness, slowness, fatigue and low physical activity defines frailty. Fried et al.’s phenotype does not take cognitive status into account. The second theory, developed by Rockwood and colleagues, describes frailty by measuring accumulated deficits across multiple systems such as comorbidity, physical function, nutritional status, and cognitive function [9]. Based on the number of deficits, it is possible to calculate a Frailty Index (FI), and a higher index indicates more pronounced frailty and predicts adverse outcomes [5].

In stroke, the modified Rankin scale (mRS) is often used for measuring pre-stroke dependency [10]. However, the scale is somewhat crude, focused on mobility, and lacks information on comorbidity and cognitive function [11]. Pre-stroke mRS is a well-known tool for assessing premorbid ability for selection of patients in stroke studies, but the relation to cognitive outcome post-stroke is fairly studied. The mRS does not take into account the heterogeneity seen in older adults, which may be better reflected by measuring frailty.

Frailty measures have demonstrated their prognostic accuracy for mortality and general outcomes in many fields of medicine, but remain to be tested for prognostication of post-stroke NCD. Studies have shown an association between frailty and cognitive impairment in a general population [12, 13], and between frailty and vascular dementia [14].

### Aims

In the Norwegian Cognitive Impairment after Stroke (Nor-COAST) study, we aimed to compare the predictive value of pre-stroke FI with pre-stroke mRS for post-stroke cognitive impairment.

Further, we explored the added value of including pre-stroke FI in prediction models for cognitive prognosis post-stroke.

## Methods

The Nor-COAST study is a multicenter prospective cohort study recruiting consecutive participants in the acute phase of stroke from five Norwegian stroke units 2015–2017. The study was approved by the Norwegian Regional Committee for Medical and Health Research Ethics (REK) North, (REC number 2015/171) [15]. Participants gave informed written consent; if unable to give consent, informed written consent was given by a family proxy. Further details are described in the protocol article for the Nor-COAST study [3].

Demographic characteristics, such as age, sex and years of education, vascular risk factors, National Institutes of Health Stroke Scale (NIHSS) score, acute stroke progression and infections treated with antibiotics, were collected at baseline [3].

Baseline brain magnetic resonance imaging (MRI) were assessed for white matter hyperintensities (WMH) and medial temporal lobe atrophy (MTA). WMH were rated according to the Fazekas scale [16]. MTA was rated according to the Scheltens scale [17].

Pre-stroke global function was described by the mRS, a seven-level scale running from zero up to six, covering the entire range of functional outcomes from no symptoms to death [18].

The trained study nurses based the assessment of pre-stroke function on an unstructured interview.

### Cognition

Premorbid cognitive status was based on Global Deterioration Scale (GDS) [19], a global measure of cognitive function using all available information from cognitive and functional tests and self-/proxy reporting [15]. By description of the behavioral characteristics of the stroke survivor given by the patient him/herself or his/her proxy, the pre-stroke cognitive state was graded by trained study nurses into stage 1–7 at the GDS scale. Patients with pre-stroke NCD were not excluded from this study.

At 3-month follow-up, cognitive function was assessed with a 30 min neurocognitive test battery, based on the National Institute of Neurological Disorders-Canadian Stroke Networks Harmonization Standards [20], validated and adapted for Norwegian participants. The following cognitive domains were assessed: complex attention (Trail Making Test A-TMT-A), executive function (Trail Making Test B-TMT-B and Verbal Fluency Test Letters-FAS), memory (Word List Delayed Recall), language (Verbal Fluency Test Category-animals), and perceptual-motor function (visuospatial/executive subtest of Montreal Cognitive Assessment) [15, 21]. NCD was defined according to the 5^th^ Edition of the Diagnostic and Statistical Manual of Mental Disorders (DSM-5) [22] criteria. Published international normative data from high-income Western countries comparable to Norway were used. Participants performing ≥ 1.5 SD below the normative mean in ≥ one of the five cognitive domains were defined as having any post-stroke NCD, as described in detail in a previous paper [15, 22–24].

Major NCD was defined as post-stroke NCD and dependency in instrumental activities of daily living (I-ADL); mild NCD was defined as post-stroke NCD without impairments in I-ADL. I-ADL was defined, according to the DSM-5, as the ability to manage own finances and handle their own medications (from a study question to participants) [15].

### The Frailty Index

The Rockwood FI score [5] is a simple calculation of the presence or absence of each health deficit, ranged 0–1, as a proportion of the total [6]. The FI contains a number of equally weighted deficits across different domains [25]. When at least 30 variables are included, FI is shown to be a robust predictor of mortality [26].

We generated the 36-item FI to measure frailty based on pre-stroke medical conditions, symptoms or problems recorded in the Nor-COAST baseline assessments [27, 28]. The FI was conducted post-hoc. All domains described in the original Rockwood FI were represented in our list of pre-specified conditions except the domain of walking speed. In some domains we had to do adjustments from the original FI, as we lacked information in the baseline data. This was the case regarding detailed function pre-stroke, and accordingly we used dichotomized mRS scores for this purpose, as described in the additional file. We also lacked information regarding the ability to walk 800 m, as we only had collected information on the ability to walk 200 m (See Additional file 1). For further details on generation of the FI, please see Additional file 1.

The FI score was computed using the coding shown in Additional file 1, Table I. For patients with available coding on at least 30 variables, the sum was divided by the number of codings. The resulting score was between 0.0 and 1.0, where a higher score represented more severe frailty. The FI is not meant to be dichotomized into frail and robust, but Rockwood and colleagues have earlier demonstrated in their work that 0.25 can be an empirical cut-off between robust and frail [29]. For a *descriptive presentation* in this study, the participants were categorized as ‘robust’ if FI was below 0.08, ‘pre-frail’ if FI was between 0.08 and 0.24, and ‘frail' if FI was higher than 0.24, in line with earlier work on frailty [29, 30]. The pre-frail state represents an elevated risk for becoming frail [30].

### Statistical analyses

We used logistic regression with cognitive impairment at 3-months as the dependent variable. Cognitive impairment was defined as a three category ordinal variable (normal, mild and major NCD). Preliminary analysis with ordinal logistic regression showed that the Odds Ratio (OR) for FI was higher for major than for mild impairment (test of parallel lines, p = 0.014). Hence, we used two sets of binary logistic regression analyses, one for each threshold; normal or mild vs major NCD (major NCD) and normal vs any NCD (any NCD).

The predictors of primary interest were pre-stroke frailty (full range FI) and pre-stroke mRS (ordinal scale). We reported the OR for frailty per 0.1 increase in FI. We chose to report OR per 0.1 unit on the scale, since the observed frailty index varies over some multiples of 0.1, and this is also the standard deviation of frailty in our study.

Note that the OR for FI and the OR for mRS cannot directly be compared, since these are measured on different scales.

We carried out a sensitivity analysis excluding the component “cognitive function” from the computation of the FI.

We measured the predictive values of pre-stroke mRS and FI by the area (AUC) under the receiver operating characteristic (ROC) curve. We estimated the added value of FI compared to pre-stroke mRS in terms of increased AUC, using the “somersd” command in the add-on package snp15_7 in Stata 16. This method accounts for the fact that part of pre-stroke mRS was included in the FI, and these are associated, as described in the Additional file 1.

The logistic regression analyses were carried out unadjusted, and adjusted for the following variables, one at a time: Age, sex, years of education, NIHSS score, acute stroke progression, infections treated with antibiotics, Fazekas score and MTA score. Missing values were handled using available case analysis, that is, in each analysis, we included all patients with data on the variables in that analysis (Tables 1 and  4). Ninety-five percent confidence intervals (CI) were reported where relevant, and we regarded a two-sided *p*-value < 0.05 to represent statistical significance. The aim in the Nor-COAST study was to include 1000 patients, which after expected drop out of about 25% was estimated to retain sufficient power for the main research questions, as described in the protocol article [3]. Except where otherwise noted, statistical analyses were carried out using SPSS 25.

**Table 1:** Baseline characteristics. *N* = 598

Demographics	
Mean/SD age, years	71.6/11.9
Female sex^a^	257 (43.0)
Mean/SD education, years	12.5/3.8
Mean/SD pre-stroke mRS^b^	0.8/1.0
Mean/SD GDS pre-stroke^c^	1.4/0.8
**TOAST classification**^d^	
Large-vessel disease	54 (10.2)
Cardioembolic disease	123 (23.3)
Small-vessel disease	120 (22.7)
Other etiology	14 (2.7)
Undetermined etiology	217 (41.1)
**Assessments**	
Mean/SD NIHSS (0–42) day 1^e^	3 /4
Mean/SD mRS at admittance^f^	2.06 /1.3
**Mean/SD Frailty Index baseline**	**0.14/0.10**
* Frailty Grade*	
* Robust*	*140 (23.4)*
* Pre-frail*	*367 (61.4)*
* Frail*	*91 (15.2)*
**Complications in the acute phase**	
Acute stroke progression^g^	*42 (7.3)*
Infection treated with antibiotics^h^	*63 (10.6)*
**MRI findings acute phase**^i^	
* Fazekas pathology*^j^	118 (34.1)
* MTA pathology*^k^	112 (32.4)

*SD* Standard deviation*, mRS* modified Rankin Scale*, TOAST* Trial of Org 10,172 in Acute Stroke Treatment*, NIHSS* National Institutes of Health Stroke Scale*, MRI* Magnetic resonance imaging*, MTA* Medial Temporal Atrophia

^a^numbers are n (%), unless otherwise specified

^b^*N* = 597

^c^*N* = 595

^d^*N* = 528

^e^*N* = 588

^f^*N* = 596 576

^g^*N* = 576 594

^h^*N* = 594 346

^i^346

^j^Fazekas pathology were rated according to the Fazekas scale[16]

^k^MTA was rated according to the Scheltens scale [17]

## Results

**Fig. 1:**
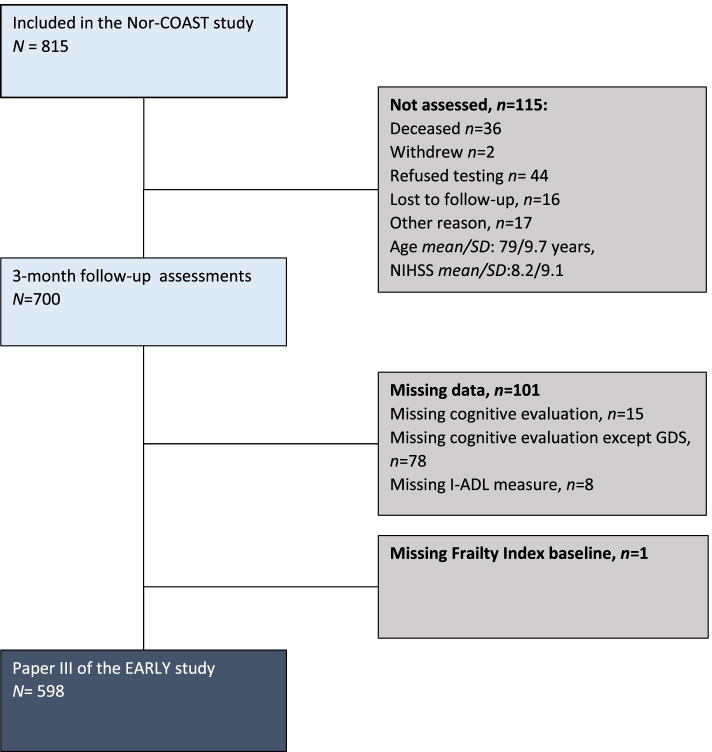
Flow chart

**Fig. 2:**
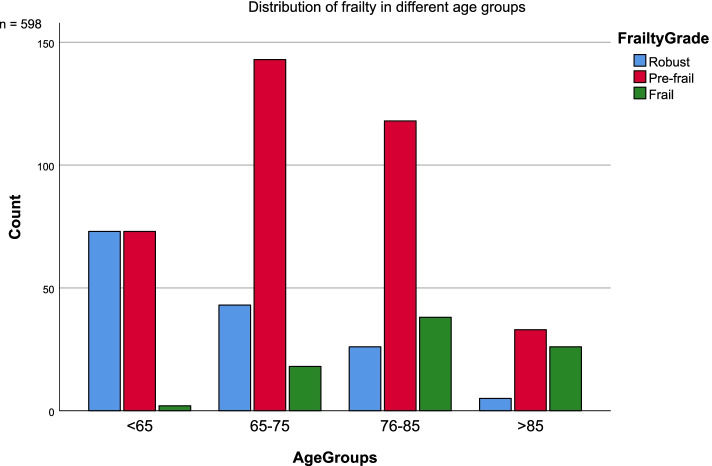
Distribution of frailty in different age groups

Of the 815 participants included at baseline in the Nor-COAST study, a total of 598 participants (43.0% women, mean/SD age = 71.6/11.9, mean/SD education = 12.5/3.8, mean/SD pre-stroke mRS = 0.8/1.0, mean/SD GDS pre-stroke = 1.4/0.8, mean/SD NIHSS day 1 3/4) had both FI at baseline and completed neurocognitive test battery at 3-months (Table 1, Fig. 1, see also Additional File 1) [15] in whom mean/SD FI score was 0.14/0.10. Of these, 140 (23.4%) participants were assessed as robust, 367 (61.4%) as pre-frail and 91 (15.2%) as frail (Table 1). The distribution of frailty in the different age groups showed an increasing number of pre-frail and frail patients in the higher age-groups (Fig. 2). The proportion with pre-stroke dementia assessed by GDS was 22 (3.7%) (Additional file 1 (Table II)).

The results of logistic regression with NCD as dependent variable and FI or pre-stroke mRS or both as predictors, are shown in Table 2. The FI, as well as the pre-stroke mRS, were strong predictors of both major NCD (OR 3.09 and 2.21) and any NCD (OR 2.29 and 1.89). When FI and pre-stroke mRS were entered as predictors for major NCD simultaneously, however, the OR for pre-stroke mRS decreased relatively more (to 1.24) than the OR for FI (to 2.67). A sensitivity analysis excluding the component “cognitive function” from the computation of the FI gave practically the same results (OR for FI vs pre-stroke mRS for major NCD; 2.97 vs 2.20, and for any NCD; 2.22 vs 1.87), see also Additional file 1 (Table III).

**Table 2:** Logistic regression analysis with NCD as dependent variable, and Frailty and mRS as predictors. *N* = 598

	OR for Major NCD	OR for Any NCD
Only one predictor at a time		
Frailty^a^	3.09 (2.45 to 3.89), < 0.001	2.29 (1.83 to 2.87), < 0.001
mRS	2.21 (1.83 to 2.68), < 0.001	1.89 (1.56 to 2.29), < 0.001
Both predictors simultaneously		
Frailty^a^ mRS	2.67 (2.01 to 3.54), < 0.001 1.24 (0.96 to 1.60), 0.10	1.92 (1.47 to 2.51), < 0.001 1.32 (1.04 to 1.68), 0.026

*NCD* Neurocognitive disorder*, mRS* modified Rankin Scale

^a^OR per 0.1 units increase in the Frailty index (FI)

The predictive values, measured as area under the ROC curve, were higher for FI than for pre-stroke mRS (Table 3). This applied to both major NCD (0.762 vs 0.677) and any NCD (0.681 vs 0.638). The difference in AUC was 0.086 (CI 0.042 to 0.129), *p* < 0.001, for major NCD, and 0.043 (CI 0.002 to 0.083), *p* = 0.039 for any NCD. Hence, the added predictive value of FI over pre-stroke mRS was larger for major NCD than for any NCD.

**Table 3:** ROC analyses. AUC (CI), *p*-value

	Major NCD	Any NCD
Frailty	0.762 (0.717 to 0.808), < 0.001	0.681 (0.638 to 0.723), < 0.001
mRS	0.677 (0.626 to 0.727), < 0.001	0.638 (0.598 to 0.678), < 0.001
Difference	0.086 (0.042 to 0.129), < 0.001	0.043 (0.002 to 0.083), 0.039

*ROC* Receiver operating characteristic curve*, AUC* Area under the curve*, CI* Confidence interval*, NCD* Neurocognitive disorder*, mRS* modified Rankin Scale

The OR for FI remained virtually unchanged when adjusting for sex, education, infection treated by antibiotics, acute stroke progression or NIHSS score at admission. Adjusted for Fazekas pathology baseline the OR was 3.44 (CI 2.47 to 4.80). Adjusted for MTA pathology baseline, the OR was 3.66 (CI 2.63 to 5.10) (Table 4).

**Table 4:** Logistic regression analysis with major NCD or Any NCD as dependent variable^*^

*N* = 598^a^	Major NCD	Any NCD
	OR (95% CI)	OR (95% CI)
Frailty unadjusted	3.09 (2.45 to 3.89)	2.29 (1.83 to 2.87)
Frailty adjusted separately for		
Age	2.52 (1.98 to 3.20)	2.01 (1.58 to 2.55)
Sex	3.08(2.44 to 3.88)	2.29 (1.83 to 2.87)
Education	2.87 (2.27 to 3.63)	2.11 (1.68 to 2.65)
NIHSS sum score at admittance^b^	3.13 (2.46 to 3.99)	2.26 (1.80 to 2.84)
Acute stroke progression^c^	3.11 (2.45 to 3.94)	2.28 (1.81 to 2.87)
Infection treated by antibiotics^d^	2.98 (2.36 to 3.76)	2.25(1.79 to 2.83)
Fazekas pathology^e^	3.44 (2.47 to 4.80)	2.17 (1.59 to 2.97)
MTA pathology^f^	3.66 (2.63 to 5.10)	2.27 (1.69 to 3.06)

*NCD* Neurocognitive disorder*, NIHSS* National Institutes of Health Stroke Scale*, MTA* Medial Temporal Atrophia

*All p-values were < 0.001

^a^unless otherwise specified

^b^*N* = *587*

^c^*N* = 576

^e^*N* = 594

^f^*N* = 346 for major NCD, *N* = 333 for mild or major NCD

^g^*N* = 346

The logistic regression analysis for any NCD 3-months post-stroke for frailty baseline showed an OR (OR per 0.1 unit increase in FI) at 2.29 (CI 1.83 to 2.87). The estimates remained approximately the same when adjusted for age, sex, education, NIHSS score, acute stroke progression, infections, Fazekas or MTA pathology (Table 4).

## Discussion

We demonstrated that pre-stroke FI is a stronger predictor than pre-stroke mRS for post-stroke major NCD. Moreover, pre-stroke frailty is a strong and independent predictor for any NCD post-stroke.

There are no general guidelines for interpreting AUC values, but the rule of thumb used by Hosmer et al. 2013 [31] indicates that an increase of 0.1 or more in AUC may be considered important, and that an AUC over 0.7 represents acceptable discrimination [31]. In sum, our study indicates that pre-stroke FI is clearly better in predicting post-stroke major NCD than pre-stroke mRS, whereas the difference is not so convincing regarding any NCD.

Different well-known risk factors and mechanisms are thought to play an important role in developing post-stroke NCD, hereby the stroke severity and the brain resilience. Brain resilience is described, partly as cognitive reserve (age, education, life style factors) and partly as brain reserve. Brain reserve is explained by individual differences in brain size and chronic brain pathological changes [32]. In our prediction model, we adjusted for these potential confounding variables operationalized as white matter lesions (Fazekas score) and atrophy (MTA score). Age is the only risk factor that, to some degree, influences the effect of FI both for developing major NCD and for any NCD. Other risk factors did not modify the effect of frailty.

An association between frailty and NCD has also been observed in non-stroke populations. The association has only to a very limited degree been studied in stroke populations, though with essentially the same results as we showed in this study [25, 33]. Mechanisms from dysfunction in multiple organ systems are discussed to contribute in the development of post-stroke NCD, and our results extend the findings of previous work in this field, by demonstrating the strong association between frailty and NCD.

Moreover, we found a high occurrence of pre-stroke frailty, with almost 80% of the population being pre-frail or frail before the incident stroke. This is in line with findings in a similar study of frailty in a stroke population [30, 34], and should be an important factor when planning for treatment, secondary prevention and rehabilitation.

The mRS is a well-established tool in stroke medicine, both for research and in clinical settings. Pre-stroke mRS assess premorbid function for selection of patients for stroke studies, but the relation to cognitive outcome post-stroke remains to be evaluated. If the mRS had shown to be equal to the FI for predicting post-stroke NCD, the introduction of another tool for stroke medicine would have been unnecessary.

During the last decade, frailty assessments have become a mainstay in geriatric medicine. Our findings imply that frailty assessments deserve their place in stroke medicine in prediction of cognition as well as overall prognosis, and potentially as a part of clinical decision-making regarding acute treatment for stroke. Future studies are needed to address the latter [35]. A full FI assessment might be time consuming, and a short version, like the Clinical Frailty Scale [7] exists, but the predictive value needs to be evaluated in stroke patients. Like the mRS, Clinical Frailty Scale might be too crude and lose predictive value compared to a full FI.

We recognize there is a debate over the definition of frailty and inclusion of cognition in the concept. Therefore, comparing the predicting value of Fried et al.’s phenotype and the FI based on Rockwood for post-stroke NCD would be of interest in future studies. Further, as patients with pre-stroke NCD were included in the study, the term “marker” for post-stroke NCD could have been more precis than “predictor”. However, “predictor” is consistent with its use in the meaning of “covariate” [36] or “explanatory variable” [37].

This study has some limitations. First, we assessed pre-stroke frailty post-hoc, based on information acquired post-stroke, partly by proxies. We used information about the ability to manage own finances in the FI score and also as an I-ADL measure for cognition. Moreover, we used the pre-stroke mRS score as a means for assessing functional status as part of the FI score, and at the same time as comparator for the FI (Additional file 1). However, the statistical analyses account for all these issues. The pre-stroke mRS was assessed by unstructured interview by study nurses, which may reduce the inter-rater reliability when compared with structured assessments [11]. The generalizability of our analyses may be more valid for patients who had experienced milder strokes, as the study population had similar baseline characteristics but better pre-stroke health and milder strokes than the non-included stroke patients [38].

A strength is that we in this study used FI for assessing frailty, which is reproducible, highly correlated with mortality, and easily calculated, as part of a structured multi-professional assessment that should be routine in every stroke unit.

## Conclusions

In this study, frailty was a stronger predictor than pre-stroke mRS for post-stroke NCD.

Measures of frailty could be a part of the prediction models for cognitive prognosis post-stroke.

## Supplementary Information

**Additional file 1: Table I.** Comprehensive Geriatric Assessment-Based Frailty Index. **Table II.** Cognitive status pre-stroke based on Global Deterioration Scale (GDS) † *N*=595.  **Table III.** Logistic regression analysis with NCD as dependent variable, and Frailty (“cognitive function” excluded from the frailty index) and mRS as predictors. *N* = 596.

## Data Availability

Study data are available from the corresponding author on reasonable request.

## Abbreviations

NCD: Neurocognitive disorder
FI: Frailty Index
mRS: Modified Rankin Scale
the Nor-COAST study: The Norwegian Cognitive Impairment after Stroke study
MRI: Magnetic resonance imaging
WMH: White matter hyperintensities
MTA: Medial temporal lobe atrophy
GDS: Global Deterioration Scale
NIHSS: National Institutes of Health Stroke Scale
TMT: Trail Making Test
DSM-5: The 5th Edition of the Diagnostic and Statistical Manual of Mental Disorders
I-ADL: Instrumental activities of daily living
OR: Odds Ratio
AUC: Area under the receiver operating characteristic curve
ROC: Receiver operating characteristic curve
CI: Confidence interval
