# Supplementary material for: Is Frailty Index a better predictor than pre-stroke modified Rankin Scale for neurocognitive outcomes 3-months post-stroke?
Source: BMC Geriatr. 2022 Feb 19;22:139. doi: 10.1186/s12877-022-02840-y (PMC8857811; doi:10.1186/s12877-022-02840-y)
Supplement: Supplementary file 1 — Additional file 1: Table I. Comprehensive Geriatric Assessment-Based Frailty Index. Table II. Cognitive status pre-stroke based on Global Deterioration Scale (GDS) † N=595. Table III. Logistic regression analysis with NCD as dependent variable, and Frailty (“cognitive function” excluded from the frailty index) and mRS as predictors. N = 596. [file 12877_2022_2840_MOESM1_ESM.pdf]

ADDITIONAL FILE 1

Is Frailty Index a better predictor than pre-stroke modified Rankin Scale for Neurocognitive outcomes 3-months Post-stroke?

Composing the Frailty Index

The different domains in the Norwegian translated FI are divided in medical history with undergroups cardiovascular and non-cardiovascular diseases and medications, functional limitatons with activities of daily living (ADL), instrumental ADL (I-ADL), Nagi-items and Rosow-Breslau, cognitive function, physical functions and nutrition.

In this study we made 36 variables out of 48 in the Norwegian translated FI, all domains except walking speed was represented. Information came directly from medical record/CRF unless otherwise specified.

The FI was conducted post-hoc, as FI was not included as a primary aim in the original study. Therefore, in some domains, we had to do adjustments from the original FI, as we lacked information in the baseline data;

For ADL it was impossible to use Barthel Index in the FI, as it was measured 7 days post-stroke in the study. Instead, we used Nottingham E-ADL for information regarding eating, for the rest we converted modified Rankin scale (mRS) pre-stroke to ADL function, where mRs pre-stroke 4-5 was set to identical with impairment/in need of help. For showering mRs 3-5 was set to be impairment. We lacked information regarding walking aids.

Coronary heart disease was defined as percutaneous coronary intervention (PCI) or coronary artery bypass grafting (CABG), and peripheral vascular disease was defined as Claudicatio intermittens.

For cognition we transformed the Mini-Mental Status Evaluation (MMSE) score to Global Deterioration Scale(GDS); MMSE 27-30 to GDS 1-2, MMSE 24-26 to GDS 3, MMSE 21-23 to GDS 4, MMSE <21 to GDS 5-7.

Under the Rosow-Bresau items domain we had to make adjusted variables: unable to walk 800 m changed to unable to walk 200 m, unable to walk up or down stairs changed to unable to walk up or down *a flight* of stairs, and we lacked information regarding heavy housework.

In the domain Physical performance the Gripth strength was defined out of max strength of both sides, three trials. This was measured post-stroke in the acute phase, but we assumed the healthy side performed as pre-stroke. We lacked information regarding walking speed. In the domain Nutritional status, we lacked albumin status.

**Additional file, Table I. Comprehensive Geriatric Assessment-Based Frailty Index**			
	**Frailty Index (FI)**		
**Domain**	***Variables used in the FI***	***Tools***	***Coding***
Medical history			
**Cardiovascular**	Angina pectoris		No=0<br>Yes =1
	Atrial fibrilation or flutter		No=0<br>Yes =1
	Congestive heart failure		No=0<br>Yes =1
	Coronary heart disease		No=0<br>Yes =1
	Diabetes mellitus		No=0<br>Yes =1
	Hypertension		No=0<br>Yes =1
	Myocardial infarction		No=0<br>Yes =1
	Peripheral vascular disease		No=0<br>Yes =1
	Stroke or TIA		No=0<br>Yes =1
**Non-cardiovascular**			
	Cancer diagnosed within 5 years or metastatic cancer		No=0<br>Yes =1
	Chronic kidney disease		No=0<br>Yes =1
	Chronic obstructive pulmonary disease		No=0<br>Yes =1
	Depression		No=0<br>Yes =1
	Fall in the past year		No=0<br>Yes =1
	Sensory impairment (hearing or vision impairment)		No=0<br>Yes =1
**Medications**			
	Use of ≥5 prescription drugs		No=0<br>Yes =1
Functional limitations			
**ADL items**			
	Need personal help for feeding		No=0<br>Yes =1
	Need personal help for dressing and undressing		No=0<br>Yes =1
	Need personal help for grooming		No=0<br>Yes =1
	Need personal help for getting in and out of bed		No=0<br>Yes =1
	Need personal help for bathing or shower		No=0<br>Yes =1

	Need personal help for using toilet		No=0<br>Yes =1
**E-ADL items**			
	Need personal help for using telephone		No=0<br>Yes =1
	Need personal help for using transportation		No=0<br>Yes =1
	Need personal help for shopping		No=0<br>Yes =1
	Need personal help for preparing own meals		No=0<br>Yes =1
	Need personal help for housework		No=0<br>Yes =1
	Need personal help for taking own medications		No=0<br>Yes =1
	Need personal help for managing own money or paying bills		No=0<br>Yes =1
**Nagi items**			
	Not writing letters* (changed from *difficulty in writing or handling small items*)	Nottingham E-ADL	No=0<br>Yes =1
**Rosow-Bresau items**			
	Unable to walk up or down a flight of stairs*(changed from *unable to walk up or down stairs)*		No=0<br>Yes =1
	Unable to walk 200 m*(changed from *unable to walk 800 m*)		No=0<br>Yes =1
Cognitive function			
**Global Deterioration* Scale** (changed from MMSE)	1-2p*		0
	3p*		0.3
	4p*		0.7
	5-7p*		1
Physical performance			
**Gripth strength**			
	male≥32.0kg/women≥20.0 kg		0
	male 26.0-31.9 kg/women 16.0-19.9 kg		0.5
	male<26.0kg/women<16.0 kg		1
Nutritional status			
**Low weigth**	BMI <21 kg/m^2		No=0<br>Yes =1
**Weigth loss**	Unintentional weight loss >4.5 kg in the past six month		No=0<br>Yes =1

Note: 36 variables out of 48 in the Norwegian translated FI, all domains except walking speed is represented

** Adjusted variable*

**Additional file 1, Table II. Cognitive status pre-stroke based on Global Deterioration Scale (GDS)** [†] **N=595**	
1*(No cognitive decline)	446 (75.0)
2 (Minimal cognitive impairment)	91 (15.3)
3 (Mild cognitive impairment)	36 (6.1)
4 (Mild dementia)	14 (2.4)
5 (Moderate dementia)	8 (1.3)
6 (Moderately severe dementia)	0 (0)
7 (Severe dementia)	0 (0)

** numbers are n (%), unless otherwise specified*

† The 7 stages can further be classified into 2 categories. Stages 1 to 3: the pre-dementia stages, stages 4 to 7: the dementia stages.

Additional file 1, Table III. Logistic regression analysis with NCD as dependent variable, and Frailty ("cognitive function" excluded from the frailty index) and mRS as predictors. N = 596

	OR for Major NCD	OR for Any NCD
Only one predictor at a time		
Frailty [1]	2.97 (2.37 to 3.72), <0.001	2.22 (1.78 to 2.76), <0.001
mRS	2.20 (1.81 to 2.66), <0.001	1.87 (1.52 to 2.67), <0.001
Both predictors simultaneously		
Frailty [1]<br>mRS	2.52 (1.91 to 3.32), <0.001<br>1.28 (0.99 to 1.65), 0.057	1.85 (1.42 to 2.40), <0.001<br>1.34 (1.05 to 1.71), 0.018

NCD = Neurocognitive disorder, mRS = modified Rankin Scale,
[1] OR per 0.1 units increase in the Frailty index (FI)

Included participants:

We included participants from the Nor-COAST study who had a complete comprehensive cognitive test battery or at least part of the battery as long as they had scored ≥1.5 *SD* below the normative data in one or more cognitive domains. The included participants should also have a frailty index baseline. Participants with communication difficulties after the stroke may have performed worse on neurocognitive tests due to communication difficulties and were therefore either excluded because they failed to complete the neurocognitive test battery, or assessed as poorer cognitive function due to the communication difficulties.
